# Suppression of malaria transmission and increases in economic productivity in African countries from 2007 to 2011

**DOI:** 10.5281/zenodo.10878649

**Published:** 2014-03-04

**Authors:** William R. Jobin

**Affiliations:** 1 Blue Nile Associates, 25558 Road N.6, Cortez, Colorado 81321, USA

## Abstract

**Background:**

To test the assumption that reductions in malaria in Africa will increase economic productivity, a correlation-regression analysis was conducted to evaluate the impact of expenditures by the US President’s Malaria Initiative for Africa (PMI), and increases in the economic productivity of countries included in the PMI.

**Materials and Methods:**

For the 12 most representative countries the per capita expenditures for malaria suppression in the 2011 budget of the PMI were compared with observed increases in per capita economic productivity. The measure of economic productivity used was the per capita Gross Domestic Product (GDP) for the period 2007 to 2011.

**Results:**

With a mean annual expenditure for suppressing malaria slightly above 1 US dollar per capita (range 0.44-3.40), there was a positive but weak correlation of higher expenditures with increased economic productivity. The correlation coefficient r was 0.5. The increase in per capita GDP in these countries over the 4-year period varied between 60 and 200 USD. The slope of the regression line and thus the ratio of benefits to cost from this programme varied slightly between ecologic zones, but the mean was 6.75 to 1. This meant that there was an increase in per capita GDP of $6.75 for every $1 invested per capita in suppressing malaria.

**Conclusions:**

The high benefits to cost ratio from the PMI makes suppression of malaria by methods used by the initiative potentially an attractive investment, at least for the near future while the biocides and drugs deployed are still effective.

## 1 Introduction

The expense of suppressing malaria in Africa has traditionally been justified by the expected improvements in human survival and quality of life, by expected national savings in health care expenses, and by expected direct benefits to economic productivity [1]. It has been widely asserted that malaria infections in African workers reduce national economic productivity through increased absenteeism and impairment of efficiency whilst at work, thus providing economic justification for expensive programmes to suppress malaria transmission on a national or even a continental scale. At a fundamental level, the relation between malaria and economic productivity was examined with the aim to determine whether widespread suppression of malaria in Africa could erase one of the factors that has retarded its development.

Because of the scarcity of data, measuring the impact of malaria suppression on economic productivity has been difficult in the past. Investigators have had to resort to complex mathematical analyses because of the lack of uniform measurements on malaria transmission and suppression efforts. Nonetheless, it was estimated over a decade ago that tropical countries that had a 10% reduction in malaria prevalence were associated with 0.3% higher annual economic growth during the period 1965 to 1990; a small but positive relation [2]. Using an econometric model, it was also very recently estimated that malaria in Uganda resulted in an annual loss of 0.8% of their per capita GDP in 2003 [3]. For Zambia it was estimated in 2005 that an improved program for treatment of malaria infections would result in a 1.8% increase in the per capita GDP, based on a ‘willingness to pay’ analysis [4].

In the current analysis of the impact of malaria suppression on productivity, the measure used to assess economic productivity was the per capita gross domestic product, expressed as ‘per capita GDP’ in terms of current US dollars and mid-year national population sizes [5]. This per capita GDP was the aggregate output of all goods and services in the countries examined, including personal consumption, government expenditures, private investment, capital and net exports, divided by the population.

Verifying the relation of malaria suppression to in-creased economic productivity is complex because of the high variability in both parameters across the several ecologic zones of Africa. Economic activities as well as patterns of malaria transmission vary with ecology - especially with seasonal patterns of temperature, rainfall and humidity, which affect the malaria mosquitoes and also affect human behaviour such as sleeping patterns. One of the reasons why malaria stubbornly resists suppression in Africa is because people are often forced to sleep outdoors due to high temperatures and humidity during malaria transmission seasons. These conditions also vary widely between ecologic zones.

An additional source of variance in economic performance is the unusually high GDPs of some countries due to their ability to extract and export oil and precious minerals. Because of its large oil fields and diamond mines, Angola had a calculated per capita GDP over $5.000 in 2013, ten times that of Tanzania which depends on subsistence agriculture and has few valuable exports [5]. The economics of oil and precious mineral exportation are largely dependent on their prices on the world market and thus independent of labour efficiency, which might be affected by malaria. Clearly the GDP data for oil and mineral exporting countries requires special treatment in a continental analysis.

In the past few years, many African countries have seen remarkable economic growth, after decades of stagnation. This has been most evident in West Africa where the end of civil wars and the establishment of stable democracies were followed by noticeable improvements since the beginning of this century. Liberia, with a population of about 4 million people had a 76% increase in its GDP between 2007 and 2011 (Table 1). This corresponds to an annual increase of 19%; strong economic growth.

**Table 1.** The 19 PMI countries in 2013, located in 4 of 6 major ecologic zones of Africa.

PMI country	Ecologic zone	Launch of PMI (year)	Population (2010) in million	FY 2011 PMI budget (million USD)
Angola^1^	Savannah	2005	13.1	31
Benin	Coast-savannah	2007	9.8	18
DR Congo^2^	Forest	2010	70.9	35
Ethiopia	Grassland	2007	93.8	41
Ghana	Coast-savannah	2007	24.3	30
Guinea^2^	Savannah	2011	10.3	10
Kenya	Grassland	2007	44.0	36
Liberia	Coast-savannah	2007	3.9	13
Madagascar	Grassland	2008	21.3	29
Malawi	Savannah	2007	16.7	26
Mali	Grassland	2007	16.0	27
Mozambique	Savannah	2007	24.0	29
Nigeria^2^	Coast-savannah	2010	152.2	44
Rwanda	Savannah	2007	12.0	19
Senegal	Savannah	2007	12.3	24
Tanzania	Grassland	2006	48.2	47
Uganda	Savannah	2006	34.7	35
Zambia	Savannah	2007	14.2	24
Zimbabwe^2^	Savannah	2011	11.7	12

^1^ Economy based on oil and diamonds

^2^ PMI activities launched very recently

But strong growth has not occurred in every African country. During this same period (2007 to 2011), Senegal had an increase of only 13%, while the increase in Kenya was 11%, equal to annual increases of only about 3% (Table 1). Nonetheless, this wide disparity in economic growth across Africa offered the opportunity to explore the influence of the various factors causing economic growth - such as malaria suppression - if an adequate base of data could be found.

**Figure 1.**
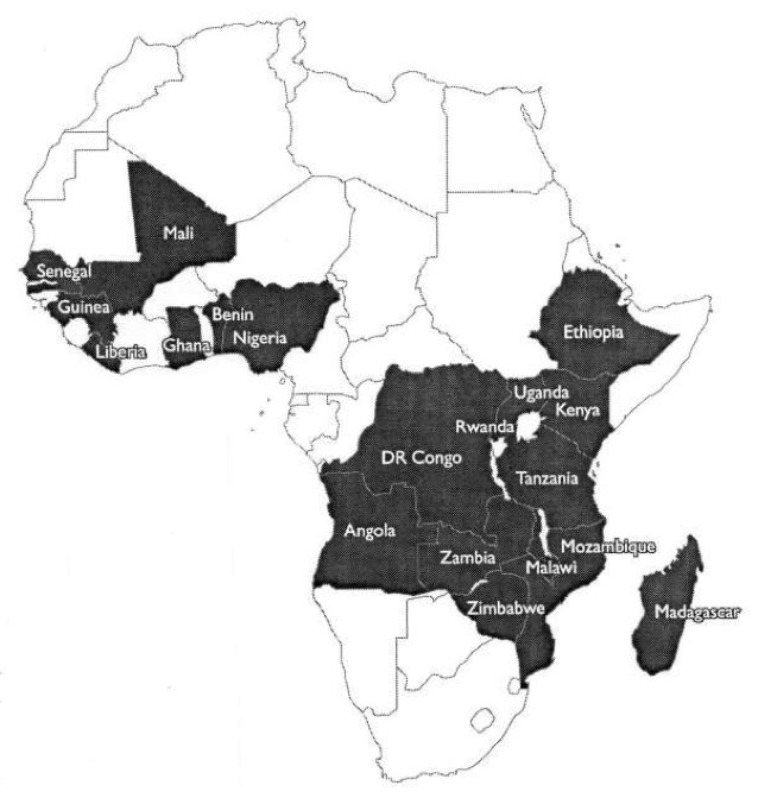
African countries in the US Presidential Malaria Initiative, 2013.

A new programme of malaria suppression has provided a new database for analysing the impact on economic development. For decades, the World Bank and other organisations have gathered widespread economic data, but the availability of data on malaria suppression has been irregular in the past. Before the turn of the century, efforts to suppress malaria in Africa were minimal and sporadic, as were the data on malaria transmission. However, in 2005 the USA established the US President’s Malaria Initiative for Africa, known as the PMI. Starting in Angola, the PMI has gradually expanded to cover 19 African countries, with an annual budget of over half a billion US dollars by 2013 [6]. These 19 countries (Fig. 1) are now showing the effects of long-term suppression of malaria on economic development. The PMI now also supports malaria suppression in Asia and the Americas as well.

Growth of the PMI in the past several years has not only suppressed malaria transmission in many countries, it has also created a growing body of experienced malariologists to help guide the fight against malaria; both Africans and Americans. Hopefully their accumulating expertise will gradually help to refine the initial crude attempts by the PMI at suppressing malaria when it began in 2005 [7]. There is evidence from the Americas and Asia that sup-pressing malaria also results in improved intellectual and educational achievement [8]. Again this is a difficult phenomenon to measure, and adequate data for its evaluation were not found for Africa during the period since the PMI came into existence.

The most direct measure of the impact of malaria suppression activities would normally be epidemiological measurements of changes in malaria incidence or prevalence, but the PMI has not yet developed a reliable system for monitoring malaria transmission in Africa. Instead they are monitoring changes in ‘all-cause mortality’, a parameter that is easily measured but which embodies large sources of variability due to the concurrent operation of many other health programmes in Africa [6]. To properly interpret the impact of the PMI on all-cause mortality, one must deduct the effect of widespread programmes in Africa to counter HIV/AIDS, respiratory disease, and the effects of vaccination programmes against childhood diseases, and of water supply and sanitation programmes, as well as specialised emergency programmes such as the mass administration of meningitis vaccines during epidemics.

A more precise measurement of the impact on malaria transmission from the expenditures on suppressing malaria by the PMI could be obtained with a carefully designed annual sampling effort, aimed at measuring malaria prevalence in standardised sentinel populations in the treated areas [9,10]. However, since such sampling does not seem to be in the current plans of the PMI - and because of the importance of establishing the impact of malaria suppression in Africa - supplemental analyses were explored in this study, focusing on the impact on economic development.

A basic economic parameter to measure the economic impact of malaria suppression was used: The per capita GDP. Measurements of this parameter are available for all African countries and have the additional advantage of being directly comparable with costs of implementing the PMI. In the long run, the economics of malaria suppression in Africa - the costs and benefits - might become as important or more important than the medical and epidemiological factors.

The strategy for fighting malaria, which has been employed by the PMI, has closely followed recent WHO guidelines, emphasising wide-scale distribution of bednets, biocides and drugs. Unfortunately, all of these are temporary measures, requiring continuous expenditures of hard currency, a considerable economic burden for the PMI. Permanent improvements in ecology, such as drainage or filling of mosquito habitats by community action groups or by government programmes, improved water management for irrigation and drainage systems, and permanent housing improvements including window screens, have been neglected by the PMI. In addition the PMI has avoided development of community-based microbial larviciding, which has been shown to be effective in urban Dar es Salaam, Tanzania, and which is used throughout the developed world [11]. Ecologic and community attacks on mosquito larvae have been the classical basis for successful malaria suppression in the rest of the world since the early 1900’s, and were originally part of the WHO strategy. This broader ecologic approach should be restored by WHO and the PMI to make their strategy more durable.

Also a warning is raised here that historically, previous programmes based solely on the use of these ephemeral methods of biocides and drugs to suppress malaria in Africa eventually failed, due to rapid development of resistance of the mosquitoes to new biocides, and the rapid development of resistance by the malaria parasite to drugs [9]. Thus although the short experience from the eight years of the PMI have indicated positive results so far, it is unlikely that this favourable situation will continue [10]. The first Global Malaria Eradication Programme of WHO and USAID - using similar ephemeral methods - lasted only about a decade.

Given the ephemeral nature of the control methods in the current strategy of the PMI, the question arises: How long will such expenditures be continued by external funders, and when will they ask that African countries take up the expenses? Thus the purpose of this study was to determine whether the expenditures for malaria suppression by the PMI correlated with a proportional increase in economic productivity in the countries protected. If there is a clear correlation, the protected countries might eventually want to cover the cost for their own malaria suppression.

## 2 Materials and Methods

To strengthen the correlation-regression analysis based on the use of changes in the per capita GDP for evaluating the impact of malaria suppression, only the most representative countries in the PMI were compared. Thus 5 of the 19 countries were initially excluded from the statistical analysis because of their atypical conditions. Angola was excluded because its economy is based primarily on oil and diamond exportation which overshadowed sectors of the economy dependent on a healthy labour force, while DR Congo, Guinea-Conakry, Nigeria and Zimbabwe were excluded because they were only recently included in the PMI (Table 1).

The possibility that factors other than malaria were affecting economic productivity at the same time as the PMI was suppressing malaria was an important consideration in reducing the 19 countries included in the PMI down to 14 for the study (Table 1). The 19 countries were originally selected for inclusion in the PMI on the basis of serious malaria problems but also because they had relatively stable and representative governments. As a result, the countries finally examined in this study had fairly similar climates, political histories, and economic bases. This did not make them identical, but did reduce many of the potentially confounding variables.

### 2.1 Ecologic zones in Africa

To further assist in understanding the impact of malaria on their economies, the countries were grouped according to the major ecologic zones of Africa, based on rainfall and predominant vegetation [9]. This ecologic grouping was used because it is likely that economic productivity - as well as malaria epidemiology and the difficulty of suppressing malaria - were related to rainfall, temperature and other characteristics of these zones (Table 1). The ecologic zones ranged from the parched desert zones of North and Southwest Africa to the humid broadleaf forest zone of the Congo River Basin, and included coastal savannah, savannah and grassland zones. In 2013, the 19 countries treated under the PMI were dispersed across 4 of the 5 zones; none were in the desert zone.

Various authors have determined that malaria is a major source of under-development in Africa, and that African ecology is closely linked to the impact of malaria. A series of papers by Sachs and colleagues [12] underlined the importance of ecology and malaria in affecting per capita income, in comparison to political and economic institutions. Independently, an evaluation of the role of the slave trade, colonialism and malaria ecology, indicated that malaria ecology mattered the most [13]. These rather startling conclusions, about the role of malaria ecology, were confirmed by the present analysis of the correlations between income and malaria suppression within ecologic zones.

### 2.2 Population considerations

Suppression of malaria has other beneficial aspects, besides increasing labour productivity. One of the most marked benefits is the decrease in deaths amongst children [14,15]. This decrease leads to rapid population growth, at least in the short term. Because the GDP and PMI expenditures were calculated on a per capita basis, the increased populations would affect the calculated numbers, although not the strength or character of their relationship. Changes in population size fluctuations were not recalculated each year - for simplicity - but the changes should be recognised if the presented figures are to be compared with those of other studies. Similar to examining correlations between GDP and malaria expenditures, a correlation analysis using data on childhood mortality versus malaria expenditures could be performed. Undoubtedly this would result in a positive correlation, but falls outside the scope of this paper.

The calculation of per capita GDP was based on the national GDP divided by the mid-year population [5]. If the national GDP of a country grows as fast as the population, then the per capita GDP stays roughly the same. But in strong economies, increase in per capita GDP exceeds population growth, providing individuals with additional wealth each year; a direct benefit from economic development. Growth in per capita GDP has exceeded population growth in most African countries in the last decade.

## 3 Results

The 19 countries in the PMI in 2013 were reduced to 14 for analysing the correlation between expenditures for malaria suppression and increases in economic productivity. The highest rate of growth in these 14 countries during the five-year period was 76%, in Liberia, which represents very strong economic growth (Tables 2 and 3). However, the lowest rate of growth was 11% in Kenya during this period, similar to the low rate of growth that many African countries experienced during the decades just after independence.

**Table 2.** Increases in per capita GDP for 14 selected African countries from 2007 to 2011, grouped in 3 ecologic zones.

PMI country	Ecologic zone	Population (2010) in million	GDP per capita (2007) in USD	GDP per capita (2011) in USD	Increase in GDP per capita in USD
Benin	Coastal Savannah	9.8	679	802	123
Ethiopia	Grassland	93.8	247	357	110
Ghana	Coastal Savannah	24.3	1090	1570	480
Kenya	Grassland	44.0	727	808	81
Liberia	Coastal Savannah	3.9	213	374	161
Madagascar	Grassland	21.3	327	390	63
Malawi	Savannah	16.7	268	365	97
Mali	Grassland	16.0	519	683	164
Mozambique	Savannah	24.0	368	533	165
Rwanda	Savannah	12.0	389	583	194
Senegal	Savannah	12.3	986	1119	133
Tanzania	Grassland	48.2	422	532	110
Uganda	Savannah	34.7	393	487	94
Zambia	Savannah	14.2	957	1425	468

**Table 3.** Expenditures for malaria suppression and increases in per capita GDP for 14 selected African countries in the PMI, grouped by ecologic zone, from 2007 to 2011.

PMI country	Ecologic zone	Population (2010) in million	FY 2011 PMI budget (million USD)	2011 per capita expenditure (USD)	Increase per capita GDP from 2007-2011 (USD)	Increase per capita GDP from 2007-2011 (%)
Benin	Coastal Savannah	9.8	18	1.87	123	18
Ghana	Coastal Savannah	24.3	30	1.23	480	44
Liberia	Coastal Savannah	3.9	13	3.40	161	76
	**Total**	**38**	**61**	**1.62**		
Malawi	Savannah	16.7	26	1.58	97	36		
Mozambique	Savannah	24.0	29	1.22	165	45		
Rwanda	Savannah	12.0	19	1.58	194	50		
Senegal	Savannah	12.3	24	1.98	133	13		
Uganda	Savannah	34.7	35	1.01	94	24		
Zambia	Savannah	14.2	24	1.68	468	outlier		
	**Total**	**113.9**	**157**	**1.30**		
Ethiopia	Grassland	93.8	41	0.44	110	45		
Kenya	Grassland	44.0	36	0.83	81	11		
Madagascar	Grassland	21.3	29	1.35	63	19		
Mali	Grassland	16.0	27	1.68	164	32		
Tanzania	Grassland	48.2	47	0.97	110	26		
	**Total**	**223.2**	**180**	**0.81**		
**TOTAL**		**375.2**	**399**	**1.06**		

### 3.1 Cost of malaria suppression measures

One of the advantages of working with the data from the countries covered by the PMI was the uniformity of the strategy for suppressing malaria used in those countries. With minor variations, each country received the same kind of treatment, although in varying degrees. Thus the per capita cost of the PMI operations did not reflect changes in strategy or methods but was more likely an indication of the effort expended. The cost of spraying residual insecticides inside houses was relatively high, but over the period of this study, from 2007 to 2011, it was assumed to be relatively constant [10]. Comparisons between countries would have been more difficult if - for instance - one country had used only larviciding, which is relatively inexpensive, and another country had relied only on indoor residual spraying that is much more expensive. Early experience with a pyrethroid insecticide for indoor spraying in Angola - used throughout Africa by PMI until resistance developed in Tanzania - indicated a cost of $7 per capita in 2010, whereas larviciding with microbial agents costs between $1-3 per capita [10, 11].

However, even though they used the same strategy everywhere, analysis of data from the PMI annual report of 2012 indicates a wide variation between countries in per capita expenditures in the 2011 budget, ranging from a high for Liberia of $3.40 to a low for Ethiopia of $0.44, with a mean value slightly over one dollar (Table 3). PMI expenditures reported on a per capita basis might differ between countries for at least two reasons. The first possibility is that more intensive efforts in applying the strategy were made in some countries than in others, such as two spraying rounds per year instead of one. The second possibility is that different proportions of the population were covered by the anti-malarial effort in different countries. This may explain why Liberia had such a high per capita expenditure. It had the smallest population of any of the countries - only 3.9 million - making it more likely that the programme protected a large proportion of the population. In the same way this might explain the low per capita expenditure for Ethiopia, which had the largest population in the 14 countries - almost 94 million people - and also the lowest per capita expenditure.

The expenditures by the Initiative were precisely documented in the annual reports of PMI to the US Congress [6]. In addition, sporadic funding for malaria suppression was supplied to some of these 14 countries by agencies other than the PMI, and to a limited extent by the countries themselves. However, the amount of these additional expenditures is not well documented. Only rough estimates were given in graphic presentations in the 2013 World Malaria Report [16].

Typical data is summarised here for Senegal, the country in the savannah zone, which had the highest per capita funding from the PMI, and for Uganda, the country in the savannah zone with the lowest per capita funding from the PMI. The contributions of other agencies were relatively small before 2011, and consisted primarily of bednet purchases and general strengthening of health services. Since bednets had short effective lives of only 3 years, and were often unused, it is not likely that these contributions had major effects on the overall success of malaria suppression.

### 3.2 Senegal

The government contribution to malaria suppression in Senegal was about $1 million per year until significant contributions by the Global Fund started in 2004, after which government contributions declined to zero. The PMI began in Senegal in 2008, but significant annual expenditures of about $20 million per year did not start until 2009. The Global Fund made varying contributions, between $1 million and $10 million, starting in 2004.

Several other organisations contributed to the fight against malaria in Senegal, primarily for purchases of bed-nets, and for community-based general health services. These include the World Bank, WHO, UNICEF, the Islamic Development Bank, the governments of France, Japan, China, Thailand and Belgium, and the US Peace Corps. In addition, assistance has been provided by faith-based and non-governmental organisations including *Medicos del Mundo*, the Child Fund Consortium, *Caritas*, the Red Cross, Malaria no More, and the Youssou Ndour Foundation. Some assistance came in through the private sector, namely the Pfizer and Total corporations. The amounts contributed by these groups were not reported, but the funds were used primarily to purchase bednets and for health education.

### 3.3 Uganda

The government of Uganda contributed about $1 million per year for several years until 2008 when their contribution had risen to about $5 million. However, the contributions stopped in 2009 and have not resumed. The PMI began contributing in 2006, and continued through 2011, with average annual expenditures of about $25 million. The Global Fund contributed about $5 million in 2004, $40 million in 2005 and 2006, with no further contributions until 2010 when they gave $150 million, averaging about $60 million per year thereafter. Their contributions were primarily to purchase bednets and for general strengthening of health services. Uganda did not report the large number of other contributors reported by Senegal.

### 3.4 Correlation between expenditure and GDP

Using the data for expenditures on malaria suppression from the PMI and for the GDP from the World Bank, a correlation-regression analysis was performed to determine r, the Pearson coefficient of correlation [17]. The most recent figures on GDP from the World Bank ended with data for 2011 at the time of this analysis, and the number of countries in the PMI was quite high by 2007, thus the analysis was performed for the period 2007-2011.

**Figure 2.**
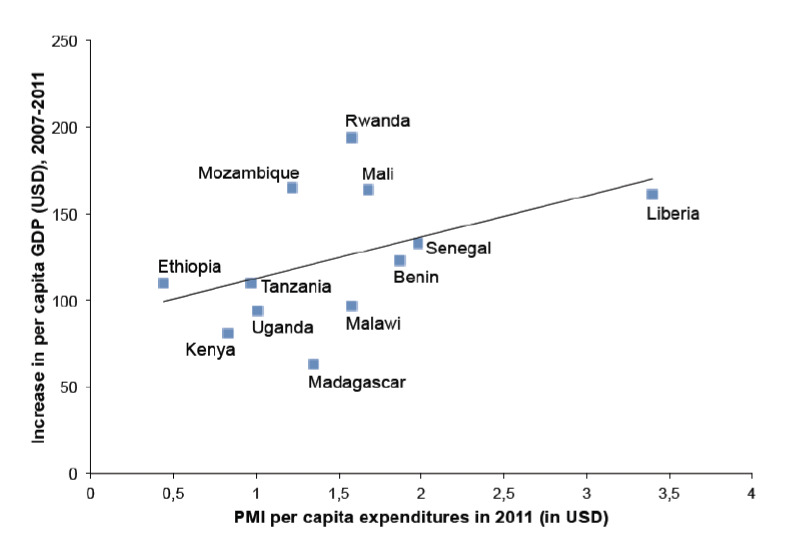
Correlation between increase in per capita GDP from 2007 to 2011 and annual expenditures in 2011 for suppressing malaria by PMI in 12 African countries.

For all 14 selected countries as a group, there was a positive but weak correlation between the amount of money expended per capita to suppress malaria, and the increase in per capita GDP (Table 3 and Fig. 2). After omitting the two obvious outliers of Ghana and Zambia, the correlation coefficient r for expenditures versus per capita GDP was 0.5.

Although most of the data points fell fairly close to the regression line, Ghana and Zambia were obvious outliers. Both these countries had increases in per capita GDP over $400, while the maximum for any of the other countries was less than $200. Perhaps the importance of copper mining in Zambia’s economy would explain its uncommon nature. Furthermore, Ghana has recently undergone a major re-calculation of its GDP, using a more recent base-year, and large revisions in the share and volume attributed to its three economic sectors of agriculture, industry and services [18]. The recalculations made in 2010 raised the GDP by about 60%, putting Ghana in a much higher development category.

Another source of uniqueness which might explain why Ghana and Zambia were outliers is the presence of their large hydroelectric installations: Akosombo Dam and Volta Lake in Ghana, and Kariba Dam in Zambia. These are some of the largest hydroelectric dams in Africa, and not only support aluminium and copper production in the respective countries, but also make it possible for the countries to export electricity. This economic activity is independent of labour productivity, whereas the average country in Africa is highly dependent on it.

There are several reasons to explain the generally low correlation for this data set. One is the uncertainty in published GDP figures, such as the major recalculation required for the data from Ghana. Another is the poorly documented efforts of private organisations in the distribution of bednets and other anti-malarial services. And finally there is the effect of sudden increases in population due to fewer malaria deaths. There are, however, simple ways to improve this correlation. Additional data could be sought to cover more than a 4-year time span, or data could be collected from more countries as they are added to the PMI. In both cases this would involve a wait of some years. However, rather than wait for more data to become available, the countries were grouped into three major ecologic zones to see if that would improve the correlation. Making this grouping assumed that the ecology might affect the success of the malaria suppression, and thus the increase in economic productivity (Table 3).

It was unfortunate for our analysis that only three countries fell within the coastal savannah zone. Also, given this uncertainty in recent GDP figures for Ghana, it is best not to base any conclusions on this correlation for the coastal savannah zone. There were 5 countries in the savannah zone, resulting in a correlation coefficient of r = 0.2 (Table 3). The slope of the regression line for these countries was equivalent to $6.25 to 1. Finally, there were 5 countries in the grassland zone (Table 3), with a correlation coefficient of 0.4. The slope of the regression line was slightly lower than for the other ecologic zones, equivalent to $5.50 to 1.

The slope of the regression line for all of the countries was equivalent to $6.75 to 1, indicating that in general the investment of each additional $1 per capita by the PMI resulted in an increase in per capita GDP of $6.75, a high return on the investment. The variation of this slope for the countries in the savannah and grassland Zones considered separately was small; $6.25 and $5.50 respectively. Separating the analyses into ecologic zones did not help in improving the correlations. The slight differences calculated for the slopes of the regression lines were probably not important either, except that their general similarities gave additional confidence in the overall strength of the relation between malaria suppression and increased economic productivity.

### 3.5 PMI’s stimulus to local economies

Expenditure of US government funds for local labour, operations and some equipment and supplies, directly stimulated the local economies and would have contributed something to national GDPs. However, the bulk purchases of drugs, bednets, biocides, spraying equipment, personal protective gear for the spraying personnel and administrative costs outside of the treated countries did not contribute to national GDPs.

Analysis of the economic data indicated that the national stimulus from local expenditures by the PMI - as well as the multiplier effects - was very small, being roughly less than 0.001% of the national GDPs. There might be important local impacts in the smaller countries, especially if a country were to consider a switch from US funding of the malaria suppression to local funding through their own National Malaria Control Programme or the Ministry of Health. In this case the loss of outside economic stimulus from USAID expenditures might be locally important, but not nationally. To illustrate this point, during the spray operations in Angola in 2010, out of the total expenditures of $4.5 million only $3 million were spent in Angola, mostly in the southern province of Huila [10]. The national GDP for Angola in 2010 was about $100 billion, thus the expenditures from the PMI amounted to an insignificant 0.003% of the national GDP. However, the operations and expenditures all took place in Huila Province, which had about 10% of the national population, and whose economy probably counted for less than 1% of the national economy - the province did not export oil. Thus expenditures of the PMI probably didn’t reach even 0.1% of the provincial GDP. For countries with small economies such as Tanzania and Liberia, the local contributions from the PMI would also have been a very small portion of their national GDP.

## 4 Discussion

Based on analysis of data from the PMI and the World Bank, the estimated increase in per capita GDP related to malaria suppression for these selected countries was much larger than the rates estimated from previous econometric exercises. While in 2005 it was estimated that suppression of malaria in Zambia would result in nearly 2% greater GDP annually [4], our analysis indicated a much higher increase; 49% in a four year period, or over 12% annually (Tables 2 and 3). Perhaps it would be more accurate if we subtracted a ‘background’ increase in GDP for these countries in 2011 - which was previously estimated as roughly 2 to 3% per annum. Nonetheless the corrected impact of malaria in Zambia would still be as high as 9% of the per capita GDP, not just 2%.

It had also been estimated that Uganda lost almost $2 annually from their per capita GDP in 2003 due to malaria [3], but our analysis indicated that the loss was much greater. For the period from 2007 to 2011 it would have been $94, equal to an annual loss of $23 before subtracting the 3% of background increase (Table 3). Thus the net loss would have been about $20 annually in per capita GDP, instead of the $2 previously estimated for Uganda.

Because the average cost of suppressing malaria to get these kinds of economic improvements was only about $1 per capita from the PMI data (Table 3), it might be to their economic advantage for African countries to organise their own programmes to suppress malaria, at least until the current drugs and biocides lose their effectiveness due to development of biological resistance by the parasites and the mosquitoes. Analysis of these data helped to clarify the costs and benefits of suppressing malaria in Africa if we made the assumption that the correlation between costs and benefits meant that there was in fact a cause and effect relationship. This issue will be explored in future studies.

## 5 Conclusions

A correlation-regression analysis of the data from 12 representative African countries on economic productivity and expenditures for suppressing malaria by the US President’s Malaria Initiative for Africa (PMI), between 2007 and 2011, indicated that there was a positive but weak correlation between the per capita expenditures by the PMI and increases in the per capita GDP within this group. The correlation coefficient r was 0.5. but r might increase with a larger set of data on the PMI, or through a more detailed analysis.

The mean rate of return on investment for suppressing malaria in these African countries was $6.75 for every dollar expended, a very high return. Although the correlation was positive, this was not enough to firmly establish a causal relation, it was only indicative. It’s poor correlation was due to unreliability in both the latest statistics on GDP, because of only sporadic data on other funding sources for malaria suppression, and because of increases in population size subsequent to the malaria suppression.
